# A hole in a piece of cardboard and predictive brain: the incomprehension of modern art in the light of the predictive coding paradigm

**DOI:** 10.1098/rstb.2022.0417

**Published:** 2024-01-29

**Authors:** Ladislav Kesner

**Affiliations:** ^1^ Center for Advanced Study of Brain and Consciousness, National Institute of Mental Health, Klecany 25067, Czech Republic; ^2^ Art History, Masaryk University Brno, Brno 60200, Czech Republic

**Keywords:** contemporary art, hyperprior, incomprehension, modern art, predictive coding, recognition

## Abstract

Incomprehension of and resistance to contemporaneous art have been constant features in the development of modern art. The predictive coding framework can be used to analyse this response by outlining the difference between the misunderstanding of (i) contemporary conceptual/minimalist art and (ii) early modern avant-garde art and by elucidating their underlying cognitive mechanisms. In both of these cases, incomprehension and its behavioural consequences are tied to the failure of the optimal prediction error (PE) minimization that is involved in the perception of such art works. In the case of contemporary conceptual/minimalist art the failure stems from the fact that the encounter results in non-salient visual sensations and generates no PE. In early modern avant-garde art, the occasional inability of viewers to recognize pictorial content using new pictorial conventions reflected the absence of suitable priors to explain away ambiguous sensory data. The capacity to recognize pictorial content in modernist painting, as a prerequisite for a satisfying encounter with such works and ultimately a wider acceptance of new artistic styles, required an updating of a number of expectations in order to optimize the fit between priors and sensations, from low-level perceptual priors to the development of higher-level, culturally determined expectations.

This article is part of the theme issue ‘Art, aesthetics and predictive processing: theoretical and empirical perspectives’.

## 1. Introduction

Over the past decade, the predictive coding (PC) theory of brain function and cognition has been increasingly used to model the neurocognitive foundations of aesthetics and symbolic forms and behaviours (e.g. [1–3]; see also the other papers in this issue). However, compared to language, music and literature, the extent to which it has been applied to the visual arts has been more modest. Moreover, unlike the case of music, no empirical study has yet been done to test aspects of PC in visual art perception. This may be a reflection of the fact that works of visual art do not possess anticipatory structures and statistical regularities comparable to linguistic or musical syntax, which seem to provide a better fit with the hierarchical structure of predictive error minimization (PEM). If it has been claimed that music offers a powerful tool for the investigation of PC in the brain [4], it is far from certain that the same also applies to the visual arts. At any rate, how best to advance the dialogue between the PC framework and visual art theory is at present an open question. In this opinion paper, I engage one possible strategy: following earlier attempts to relate the cognitive neuroscience of predictive perception to the interpretation of a specific work of art or to a reconsideration of an art-historical concept [5,6], this paper zooms in on the explanatory potential of PC *vis-à-vis* a particular aspect of art production and consumption. Specifically, I propose to leverage the PC framework to elucidate the complex issue of why and how people fail to meaningfully connect with modern and contemporary artworks.

This exploration does not require or indeed aim to provide a comprehensive model of PC in the visual arts and—given the space available here—it has some inherent limitations. It employs the basic tenets of hierarchical PC, but it discusses only perceptual inference (leaving aside the role of active inference), and it admittedly remains somewhat coarse-grained and removed from the rigors of the computational models of PEM. At the same time, the focus is mostly on the role of perceptual recognition/identification in pictorial understanding, rather than the entire process of meaning-making. The specific examples discussed are nevertheless representative of responses to modern and contemporary art generally.

The question that may naturally be asked is what is to be achieved by transposing PC thus into the domain of art history. For art theory, long dominated by language-based and social constructivist theories, the PC framework may provide a fresh incentive to revisit some unresolved issues that have not been well served by existing theoretical approaches. This could eventually lead to the realization that some complex socio-historical phenomena of long-standing interest—such as incomprehension and appreciative failure *vis-à-vis* art, or the problem of the historicity of vision—might best be explored by linking a cultural macro-perspective with neurocognitive models of art perception, couched in the terms of the PEM framework. In a more general sense, if understanding a work of art always requires some form of implicit inference on the part of the viewer about the hidden cause of the work's visible form, the predictive and inferential mechanisms of the human mind—all the way down to neural hermeneutics—should not lie outside the scope of art historians' and theorists' attention.

On the other hand, PC theorists have made bold claims about PC as the unifying principle of brain function, cognition and action, and they seem confident that the PEM framework is suitable for modelling cultural phenomena ([7–9]; but see [10]). However, it can also be argued that the explanatory potential of such abstract models is rather limited and that the next step is to examine the role of PEM through specific case studies of cultural–historical events and processes, defined by the humanities. What follows is an attempt to move in this direction, and it points to two central challenges for the PC modelling of culture: first, understanding how the brain as a hierarchically organized inference machine links lower levels of perceptual inferences to high-level cultural expectations; and second, determining whether single-person PEM can be transposed to the level of collective cultural phenomena, such as mastering a new language of art.

## 2. The incomprehension of contemporary art

Incomprehension and a resistance to contemporaneous art have been constant features of the development of modern art since its inception in the second half of the nineteenth century. While the public's reactions to modern art are always context-specific, it nevertheless makes sense to ask whether there are any general psychological and cognitive mechanisms underlying this kind of response. Among the many historically concrete instances of the incomprehension of art in the twentieth and twenty-first centuries, two types can be discerned that are particularly common, and the PC framework can be productively applied to explain the difference between the two and some of their underlying causes.

The first type relates to the art of the past few decades and to the different forms of conceptual and minimalist art in particular, as the following example shows. In 2013, the Chalupecký Prize, an annual award for the best work by a Czech artist under the age of 35, was awarded to the already well-established conceptual artist Dominik Lang for his installation *EastWest.* Outrage unfolded soon after, prompted by a widely circulated article published in an online magazine devoted to contemporary art. The piece was written by a young art history student, who stated:

‘The jury awarded the first prize to an artist who made two round holes in a cardboard wall, which covers the windows of the Trade Fair Palace [the seat of the modern art collection of the National Gallery in Prague]. Thanks to these holes the visitor will purportedly be able to observe the red circle of the rising and setting sun. During the day, however, what remains to be seen is just a hole in cardboard … …' [11]

**Figure 1.**
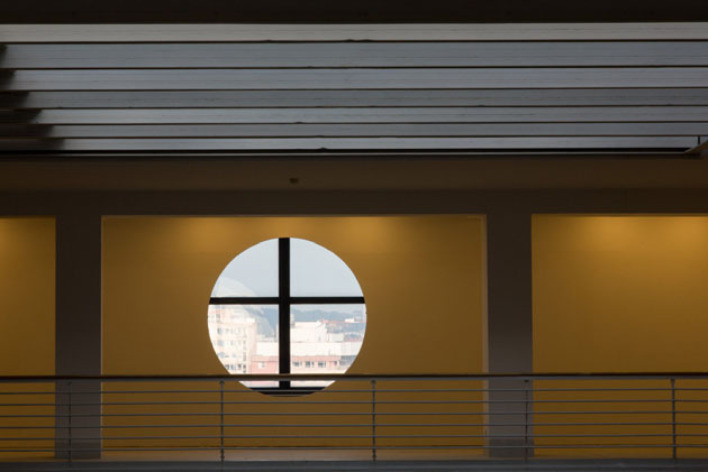
Dominic Lang, *EastWest,* 2013. A view of installation. Image source: archive of the author.

In the ensuing discussion, the vast majority of responses supported her point of view (figure 1).

This particular affair has some famous precedents, such as the scandal that erupted in 1976 after the Tate Gallery acquired Carl Andre's *Equivalent VIII*, a minimalist sculptural object consisting of 120 identical bricks arranged in two layers into a six-by-ten rectangle. The venerable art institution was ridiculed by the general public and the art establishment alike and accused of buying a ‘showy work which may well be regarded in a few decades as trash' ([12], p. 188). This kind of resistance and rejection is hardly exceptional; on the contrary, it continues to be a fairly common reaction to much contemporary art.^1^ Importantly, such reactions are by no means limited to ‘naive' viewers, as experienced art critics and even artists themselves have likewise been seen to point out that conceptual and neo-conceptual art—and contemporary art more broadly—is boring, trivial, unintelligible and untranslatable (e.g. [14–19]). Empirical research has also provided some evidence that many museum viewers dislike and reject conceptual art [20].

The traditional way of accounting for such reactions is to argue that progressive art challenges the prevailing concept of taste. This was exactly the response of the curator responsible for purchasing Andre's bricks: ‘…whatever has later been seen to have been vital in a period's art has usually been unacceptable to established taste in its own day' ([21], p. 762). A more sophisticated argument is that modern art alienates viewers by defying established meanings and conventions, and special sensibilities are required to decode and appreciate such art [22]. This idea is explored in Pierre Bourdieu's sociology of culture, which links a viewer's alienation to a lack of cultural capital, suggesting that the person has not mastered the perceptual codes that one needs to know in order to decipher a work of art ([23]; [24], pp. 216–217). The negative reactions to contemporary art are then often explained as stemming from a lack of aesthetic education [25].

The acceptance or rejection of art is a sociological phenomenon, but it is rooted in the psychological and cognitive mechanisms of the observer. Cultural competence, after all, is largely a cognitive competence [26]. If that is the case, PC can perhaps provide a suitable conceptual framework for examining the incomprehension and hostility with which contemporary art is sometimes received. The basic tenet of hierarchical PC states that the brain compares prior beliefs or hypotheses with sensory data and whenever incoming data violate these predictions, a prediction error (PE) is generated to update the predictive model at higher levels. Art perception generates PEs, and they trigger the interpretive process, which may in itself be rewarding [27]. A meaningful encounter with visual art starts with visual sensations, and given the evolutionary constraints on our visual system this inevitably entails a process of recognition and interpretation of a visual (i.e. pictorial) scene [28,29] Nevertheless, a deeper, more satisfying experience of a work of art requires that viewers are able to move beyond the ‘rush to the object', i.e. the recognition stage, and it hinges on their implicit ability to create and temporally sustain a productive flow of predictions across hierarchical levels, from low-level sensory to abstract semantic and conceptual levels [5].

If we accept the claim that art taps into the predictive capacities of the brain, we see that in the two examples of a rejection of art described above, the work fails to do this. In the case of Lang's installation and Andre's object, the sensory–perceptual experience ends in easy recognition and identification. A hole in a piece of cardboard is instantly seen as just that: a hole in a piece of cardboard. A block of bricks is explained away as a block of bricks. Correctly predicted states are not needed for the actualization of priors and, therefore, are evaluated as non-salient and dull. Minimal (or no) PEs are generated in the process of recognizing/identifying an object and if there are any, they are instantly minimized. Once the minimization of PE at the level of object recognition is concluded, there is no motivation for any further active exploratory behaviour—for prolonged observation and engagement with the work. On an experiential level, such work is found to be obvious, banal and trivial. Whatever meanings and intentions the work is said to have, in what are often tortuous explanations expressed in thick theoretical jargon, they tend to be divorced from sensory evidence. Carl Andre described his work as conveying a sense of ‘wading in bricks' and like ‘stepping from water of one depth to water of another depth' ([21,30], pp. 150–59) and on the meaning of Lang's installation one critic noted that visitors could not in fact see a sunset or sunrise through the circular aperture because visitor regulations prevented them from doing so, hence the work served as critical commentary on the functioning of public institutions. The hidden cause behind the work cannot be inferred from what it affords visually. The hermeneutical cycle, in which an encounter with a work of art is supposed to trigger a complex interplay of predictions that span every level within the hierarchical structure of the mind/brain, from sensory/recognitional to high-level semantic predictions, is not activated.

Aversion or hostility to such works then may well be a secondary response that results from the viewer's implicit appraisal of the experience of indifference and boredom. On such an account a PC-inspired explanation is indeed not incompatible with psychological theories about the threat of meaninglessness [31]. In some viewers, the appreciative failure may originate in their being unaware of the claims that it is the nature of conceptual art to be anti-visual ([32,33], p. 6) or even intentionally ‘unaccountable' and defying comprehension ([34], pp. 88–91). In this way their ontological expectations of what art is supposed to afford are frustrated [35]. In other viewers, on the other hand, even their awareness of what conceptual art is supposed to be may still not override the deeply-ingrained expectation that a work of plastic art, in order to be found rewarding, needs to offer viewers the opportunity for a perceptually rich encounter; that it should in some way challenge the viewers' perception, and if it provides no great perceptual challenge then it should at least still provide some incentive for motivated seeing that prompts feelings, thoughts and self-reflection.

## 3. Incomprehension in early modernist avant-garde art

We now turn to another instance of incomprehension that involves numerous well-documented cases of dismissive reactions to early-modern avant-garde art — typically Cubist, Expressionist and abstract. I shall argue that although in behavioural terms they are similar to the negative reactions to conceptual art, they are different from the cases just discussed, and the PC framework may provide a way of accounting for this difference. An exploration of late nineteenth and early twentieth century art criticism and journalism reveals an endless string of dismissive judgements and derogatory comments on contemporary art, in which phrases like ‘bizarre anatomies', ‘deformations' and ‘contortions' abound, and such art is repeatedly declared ‘ugly' and ‘incomprehensible' (see, e.g. [36]).

**Figure 2.**
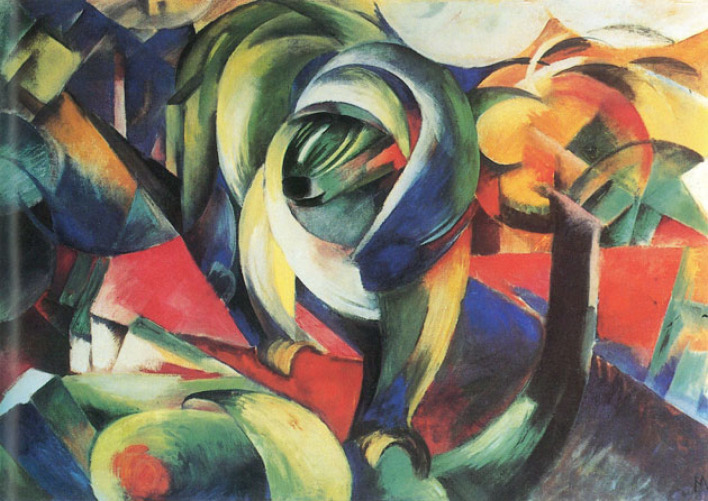
Franz Marc, *Mandrill*, 1913. Image source: Wikimedia commons.

But rather than quoting from these sources, let us consider an insight offered by the renowned art historian Ernst Panofsky. In 1931, Panofsky published one of his most significant papers, a formulation of a method for describing works of art, which formed the basis for the famous concept of three levels of meaning that he developed later. Importantly, the text contains a brief passage on contemporary art, in which Panofsky eschews any value judgements, but makes an observation of great pertinence for the present discussion:

*We only have to imagine a painting by Franz Marc instead of the Grünewald—for instance the Mandrill in the Hamburger Kunsthalle—to realize that, while we might have all the concepts to uncover the phenomenal meaning at our disposal, it is not always possible simply to apply them to the artwork in question. **In banal terms, it is not always easy to recognize what is portrayed in the picture.** We may know what the kind of monkey called a mandrill is, but in order to recognize him in this picture we have to be tuned to the principles of expressionist representation which govern the design here. **In 1919, the people of Hamburg were unable to identify the object painted by Franz Marc because they were not yet familiar with the representational principles of expressionism;** ……. it is apparent that **to grasp even the most mundane of experiential conceptions or features in a picture**—and hence offer an appropriate description—depends upon familiarity with the **general representational principles which govern its design**, that is, an awareness of stylistic form which can only be acquired by a sense of historical situation'* ([37], p. 471; emphasis added; figure 2).

Other accounts suggest that, once again, even the most qualified viewers, such as artists themselves, have been in such a predicament. Thus, Wassily Kandinsky reminisced about the confusion he experienced on seeing Claude Monet's *Haystack* in his youth: ‘That it was a haystack, the catalogue informed me. I did not recognize it. I found this non-recognition painful. … I had a dull feeling that the object was lacking in this picture'. ([38], vol. 1, p. 363) Other similar instances could also be cited. Panofsky himself does not say anything about those ‘people of Hamburg' being averse or hostile to modern art as a result of their not being able to recognize the painted object, but many other critical sources of this period make this explicit link: avant-garde art is dismissed and ridiculed for the incomprehensible way in which it portrays things and for its blunders, confusions and absurdities.

‘Representational principles', or the ‘rules of representation' mentioned by Panofsky, have been extensively analysed in art historical writings. With no space here to discuss them in detail, suffice it to say that such principles include modernist formal innovations in the constitutive elements of paintings, such as the abolition of perspective and lighting, the atomization of the picture's surface into separate brushstrokes, the reduction of the picture's fictive depth, the dissolution of the picture into texture, the accumulation of similar units of sensation, the geometric stylization of volume, etc. These stylistic and formal innovations, which became increasingly apparent in the visual arts during the last quarter of the nineteenth century, constituted an updating—and in some cases a direct contradiction—of the pictorial principles that audiences were attuned to in premodern art.

How then can we account in the terms of PC for what happened when people began to encounter paintings such as Franz Marc's *Mandrill*? In both natural vision and art perception, a subjective percept involves making the best interpretation of sensory input based on hypotheses and prior knowledge. According to PC models of visual recognition, reciprocally connected cortical areas engage in a dynamic process in which predictions are modified according to incoming sensory input until a higher-level region is able to arrive at a reasonable approximation of the incoming stimulus [39–41]. Previous experience and perceptual expertise generate a distinct set of expectations that determine the interpretation of the image. As presciently formulated by Albert Gleizes and Jean Metzinger: ‘To discern a form is to verify it by a preexisting idea' ([42], p. 425). When the ‘people of Hamburg' first encountered Marc's *Mandrill* in 1919 (or when the young Kandinsky first saw Monet's *Haystack*), the encounter resulted in PEs, irreducible by existing priors.

To summarize the two kinds of incomprehension of art outlined here so far: in the first case, exemplified by Lang's installation or Andre's object, the failure of some contemporary art to incite any interest on the part of the audience may stem from the fact that the encounter results in non-salient visual sensations and generates no PE. Hypotheses about the meaning of such works are not linked to sensory evidence, i.e. the observable features of the artwork. In the case of the perception of Cubist, Expressionist, or abstract art, by contrast, exemplified by Marc's *Mandrill,* the sensations evoked in contemporaneous audiences defied their predictive capacity at several levels. At the level of object recognition, the sensory data were too noisy to be successfully minimized given (i) available perceptual priors for object recognition and as a result could not coalesce into a meaningful percept, and (ii) the high-level cultural expectation that recognizable objects can be seen in pictorial compositions (see §4), which in most viewers at that time had not yet been dampened by the emerging anti-naturalistic rhetoric of modernist criticism and theory or by sufficient exposure to new art styles.

Common to both kinds of misunderstanding is the inability of the viewer to initiate and sustain a productive cycle of hypothesis testing and PEM driven by curiosity, which characterizes the hermeneutic act of viewing and meaning-making, albeit for exactly the opposite reason. Both negligible error (in the case of the ‘hole in a piece of cardboard') and irreducible error (in the case of *Mandrill*) translate into forms of negatively valenced affect, such as confusion, anger and boredom [43,44], supressing curiosity and further exploratory behaviour and leading to avoidance.^2^ This scenario squares well with earlier observations that much of the discomfort that people experience in their encounters with art stems from an uncertainty as to what one is supposed to do in order to appreciate it, an inability to engage in meaningful interaction ([45], pp. 189–191; [46], p. 83).

Crucially, the dynamics behind the development of the two instances of art incomprehension discussed so far differ. Four decades elapsed between the case of Andre's bricks in the 1970s and Lang's holes in 2013. Thus the misunderstanding of and negative attitude toward much contemporary art appears to be resistant to the quick accommodation to the ‘shock of the new’ described by art historian Leo Steinberg, who argued that the discomfort, bewilderment, anger, or boredom that people feel when confronted with an unfamiliar new style is short-lived, and no art remains uncomfortable for very long [47]. New artistic production along these lines is often met with indifference or is rejected by many viewers and even professional art critics. Such continued indifference to and dismissal of much contemporary art implies that the expectation that there should be something interesting, challenging and rewarding to be perceived in a work of art continues to hold sway over audiences. This expectation has clearly not been supressed by the cumulative experience of several decades of exposure to conceptual and minimalist art and their theoretical infrastructure. The case of avant-garde modernist art, however, is different, for within the same time span of half a century, the hostility and aversion to it have gradually given way to general acceptance and appreciation, and this form of art has been fully absorbed into the collective cultural consciousness.

Art works at the beginning of the twentieth century did not in most cases attempt to eliminate recognition entirely. Instead they sought to defer it and render it fluctuating and problematic [48,49]. Audiences have gradually absorbed the fact that modern art abounds in perceptual ambiguities and visual indeterminacies, which tax cognitive and perceptual routines and may result in an unusual state of awareness of one's act of meaning-making. The process of coming to terms with art that was initially deemed inaccessible was being highlighted by some commentators at the same time when this art was emerging. In their book on cubism published in 1912, artists Albert Gleizes and Jean Metzinger already suggested that encountering difficult art may be rewarding:

*A great charm results from the fact that, when the object has been truly transubstantiated, the most practiced eye experiences some difficulty in discovering it. The picture that opens up only slowly seems always to be waiting for someone to question it, as if it held an infinite number of responses to an infinite number of questions … .Yes, there is a great charm in not distinguishing at first contact the individuality of the objects … .* ([42], p. 430). The assumption that genuine art must engender active spectatorship and requires work then became commonplace in twentieth century art criticism and philosophy of art [50,51]. Such views have finally been vindicated by contemporary research, which confirms that challenging art requires deliberate cognitive activity, and mastering the ability to understand ‘disfluent’ art may ultimately be pleasurable because it is intrinsically rewarding [27,52–54].

## 4. How to recognize a mandrill

Whereas the people of Hamburg in 1919 could not recognize the mandrill in Marc's picture, viewers today would presumably have no trouble agreeing with the commentator who recently wrote:

The powerful ape is composed of all the colors in the chromatic spectrum, and it seems as if it is willingly distinguishing itself from the other colors and forms that surround it. One is able to clearly make out a head with a long snout and a mop of hair. Moving downward there are two arching arms that terminate in two hands gripping two objects as the mandrill makes its way through its environment [55].

How, then, are we to account for (a conceptually simplified) distinction between the ‘state of non-recognition' experienced by the people of Hamburg in 1919 and the ‘state of recognition' among art-loving audiences today? Specifically, what had to change in the architecture of the predictive system for audiences to recognize an ape in Marc's painting (or objects in Cubist paintings) and to find viewing such difficult artworks as a source of enjoyment rather than bafflement and irritation? Interestingly, Panofsky himself noted in his paper that understanding new forms of avant-garde art depended on the ‘subconscious habituation of new forms of visual expression' ([37], p. 471). In a similar vein, Bourdieu wrote that ‘the unconscious mastery of the instruments of appropriation, which are the basis of familiarity with cultural works is acquired by slow and imperceptible familiarization, a long succession of “little perceptions”' ([24], p. 228)). In general terms, the path to recognizing representational content in modernist works of art has been forged by perceptual learning [56,57]. It has long been acknowledged that members of specific communities or aesthetic cultures exercise learned perceptual and cognitive capacities [58].

The expertise involved in recognizing a novel pictorial scene stems from an updating of a number of the expectations required to optimize the fit between priors and sensations. Expectations or predictions in perception can be usefully divided into structural and contextual, with the latter being modulated or learned by recent experience and training [59–62]. The problem of recognizing representational content in modern art differs for each style and to some extent even for individual authors. In the specific case here, the identity of the real-world objects in *Mandrill* is uncertain and fluctuating. One major source of difficulty in correctly identifying the central object is the de-differentiation of the figure/ground distinction, as the shapes of the elements in the landscape echo the animal's contours, and the abstracted forms are seen emerging out of a dense patchwork of vibrant colours. For viewers of modern art, the expectations related to figure–ground segregation—a fundamental process by which the visual system segments a scene into figures and background [63]—needed to be adjusted or replaced by a short-term contextual prior that in modern paintings the figures may to varying degrees blend into the background, to the point where the figure–ground distinction is almost entirely eliminated.

Hierarchical object recognition proceeds in multiple stages. The construction of a subjective percept (in our case, the image of an ape in a forest) thus involves an iterative cycle of predictions and PEs generated at multiple stages, along with a continuous revision of perceptual hypotheses. At low-level perception, predictions are involved in the process of grouping local elements (lines and edges) into coherent shapes [64]. Higher up, predictions pertain to object categories, with the shapes of individual objects acting as cues that facilitate the recognition of more complex objects or scenes. Finally, it is well known that context enables both the semantic and form-based facilitation of visual object processing [65–67]. Objects found in their typical contexts are recognized more accurately and rapidly than in an incongruent scene [68,69]. In the case of our painting, mutual contextual facilitation plays out in the recognition of the ape (object) and the forest (scene). In actual perceptual encounters, context-dependency often means that higher-level processing extracts contextual information that drives specific predictions, which are fed back to modulate the low-level aspects of perceptual processing [70]. Identification of the scene may thus drive the predictions relating to the identity of the object (ape), which in turn predicts its component features (e.g. snout), and these are instrumental in determining low-level pictorial features.

At this point, an objection might be made that the focus so far in this article has been entirely on perceptual recognition and identification of pictorial content, whereas since the late nineteenth century most artistic styles were eschewing or ignoring any mimetic ambitions. But even if an artistic intention is not concerned with the challenges posed to viewers at the level of perceptual recognition, the meaning-making process still starts with an initial perceptual analysis and classification [71], and it is grounded in the viewer's ability to grasp somehow the sensory evidence that he or she encounters. However, in the early modernist period, the challenge to viewers of learning the new ‘language of form' [72] concerned not just the construction of coherent precepts, but increasingly also the ability to appreciate the formal features and facture of a work of art and to generate new predictions both about its intrinsic meaning and effects and about the meaning and value of the experience itself. This implies that a meaningful and rewarding encounter with new art required an updating of a range of higher-level—'cultural'—expectations, to which we now briefly turn.

## 5. Culturally determined priors

According to the PEM theory of brain function, predictions are multilevel and hierarchical. Predictions at the lower level of the cortical hierarchy are under the control of evolutionarily-selected, more abstract and fundamental expectations, sometimes called overhypotheses [73] or hyperpriors [74–76]. These may be innate and biologically hard-wired. However, some high-level priors are formed and transmitted through participation in culture, in material and symbolic systems [77,78]. A likely candidate for the central, enabling cultural prior that operates in art perception is general pictorial competence and the capacity of seeing in/as—the ability of the human observer to see a certain object in a depictive configuration on a pictorial surface, to see ‘through' the depiction to its referent [5]. As interpretations of prehistoric archaeological finds suggest, this capacity is the result of a long evolutionary process that started at the latest around 100 000 YBP, but probably much earlier [79,80]. There is also an ontogenetic component to this capacity, as it has been demonstrated that pictorial competence is partly acquired de novo in human infants [81,82].

In visual systems, hierarchical predictive processing explores and depends on regularities in the physical features of the world, which allow an organism to form prior expectations. Owing to their static nature, these prior expectations are learned over long timescales, whereby they become encoded in the tuning properties of the sensory cortices. Ever since the beginning and gradual proliferation of depictive activities, which themselves made the visual environment more complex, visual systems have also had to explore in parallel the regularities and changes in the system of depiction, i.e. changing representational conventions. The general ability to see objects in images is always realized in and through specific, culturally determined representational systems, which create specific local horizons for the production of art and the consumption of images. In art history, these have been famously dubbed ‘visual strata’ by Wölfflin [83] or the ‘period eye' (a manifestation of a broader cognitive style) by Baxandall [58]. Such contextual, culturally determined expectations, which are instrumental in the perception of evolving artistic forms and styles, modulated and sometimes contradicted the earlier evolutionary priors of veridical (everyday) perception.

Without embracing the popular, but simplistic, notion that modernity brought about a categorically new kind of perception [84,85], it could be postulated that specific contextual cultural priors have gradually formed that facilitate the perception of newly emerging art and enable viewers to make sense of it and appreciate it. Since Impressionism, viewers of modernist paintings of various styles have been sensitized to the need to pay attention to the process by which an image emerges from a surface, to the ways in which the representational content materializes from a vast collection of local pictorial incidents and out of the painter's depictive activity [86,87]. There thus emerged in early modernism an overarching expectation that a work of plastic art will challenge the viewer's perceptual capacities and may require the viewer to make an effort to recognize what is depicted in the work, and that recognition/identification may not be always resolved, and that this taxing of one's perceptual and cognitive routines might ultimately prove rewarding. Consequently, a number of more specific expectations involved in visual art experience needed updating or revision. For instance, while viewers of western paintings have the culturally ingrained expectation to find depth cues in two-dimensional images that suggest virtual space in the pictorial space [88], this prior has increasingly come into conflict with the modernist flattening and fragmentation of pictorial space, as brilliantly embodied in Marc's *Mandrill.* Moreover, the expectation that art is challenging, to the point where the depicted objects cannot easily be recognized in a visible pictorial configuration, in fact runs counter to, or even directly clashes with, an evolutionarily earlier perceptual prior according to which two-dimensional images convey the appearance of three-dimensional objects. However, the incomplete resolution of PE at the level of pictorial recognition will often be taken as evidence of something that needs to be explained by higher-level and more abstract hypotheses, thus ushering in a productive cycle of PEM (for an initial case study see [5]).

At the same time, the self-referentiality of modernist works of art and the privileging of the artistic medium and process over the representational content in many works shifted the expectation about the goals of a meaningful pictorial experience away from grasping the depicted subject and its associations and toward appreciating the pictorial form and facture and the possibility of inferring their generative patterns (such as the artist's emotions or personality). As modern and contemporary art have been increasingly characterized by a move from the perceptual to the semantic and abstract levels of hidden causes behind a work of art, some viewers, at least, have learned to accept the fact that works of art do not need to provide any sensory gratification or perceptual challenge and can operate on a purely conceptual level. Consequently, to see a hole in a piece of cardboard for what it is can nevertheless instantiate a process of inferring hidden causes from this kind of non-salient and non-challenging visual sensation.

In sum, the expectations that are instrumental to a successful encounter with modern art developed within a few decades through a loop of mutual feedback with the emerging stylistic and conceptual innovations of twentieth century art. These new expectations modified some of the perceptual priors that are involved in the perception of pre-modern art. The above-mentioned priors were generalized across different art styles, but some contextual predictions must have been formed in response to the idiosyncrasies of particular styles of art or perhaps even individual artistic styles. While the focus here so far has been on perceptual inference, we should note that the evolving perceptual understanding of modern art styles likely depended also on another strategy of PE reduction—namely, an epistemic active inference [89]. Although there is no empirical evidence to directly support it (but see [90] for an illuminating case study), it can be reasonably surmised that encounters with modern art styles elicited changes in attentional allocation, specifically in the patterns of eye-movements while studying a painting, which facilitated a better fit between the sensory input and predicted states.

## 6. Some outstanding issues

Psychological research has long established that people prefer medium levels of sensory uncertainty [91], and, in the context of art perception, a medium stimulus complexity [92]. PEs can trigger curiosity and exploration if one feels that one has the capacity to resolve them. A relatively high degree of sensory uncertainty (large PE) can be pleasurable if one possesses the skills necessary to reduce it and ultimately arrive at some solution to a perceptual problem [43,93]. In an ongoing, temporally extended perceptual encounter, such as that afforded by a museum visit, an optimal challenge, i.e. one slightly above the perceiver's ability, is one that involves acceptable and pleasurable error reduction dynamics [44,94]. The dynamics of perceptual learning in art (as in other fields) is thus intimately linked to reward and motivation for further exploratory behaviour through further encounters with art [27,95,96]. The acquired perceptual–cognitive competence to understand modernist art styles hinged on the updating of a number of specific priors, some of which were just described, as well as expectations about the error reduction dynamics [44] involved in encounters with new art. The question that then needs to be answered is how the optimization of the perceptual and active inference required to understand modern art and turn the encounter with it into a rewarding experience spread within just a few decades from a narrow circle of cognoscendi to a much larger swath of the viewing public? In other words, can the PC framework explain the social dynamics behind the shift from non-recognition to recognition of *Mandrill*, to shed light on the ‘epidemiology of expectation updating’ that was necessary for modernist artwork to be widely accepted and appreciated? By way of conclusion, two outstanding problems can be briefly mentioned.

First, it is well-established that structural and functional changes in the perceptual system that arise from learning result in alterations to the perceptual experience [56]. Such perceptual expertise is underpinned by experience-dependent synaptic plasticity [97–99]. However, there is currently no consensus on how precisely the mechanisms of synaptic plasticity relate to predictive processing (see, e.g. [100,101])—a subject that, in any case, is beyond the scope of this paper. A puzzling question that remains is how the sort of perceptual–cognitive expertise required for a satisfying encounter with modern art could have developed so quickly. It is known that perceptual expertise, such as that possessed by radiologists, ornithologists, or some athletes, is acquired by years of practice and training. It requires the motivation to spend long hours of study in one domain, practicing and developing one's knowledge [102–104]. Even accepting that the challenge to understanding an image in these contexts is different from the one involved in the perception of art, it is still notable that the expertise required to appreciate modern art seems to be acquired much more effortlessly. Apart from perhaps artists and a handful of critics, no one in the audience of art lovers has spent thousands of hours looking at avant-garde art, and any of them could at best have seen a few exhibitions in a year.

Second, attempts have been made to extend the PC (and free energy minimization) framework to the modelling of large-scale socio-cultural phenomena. Notions of niche construction, cultural affordances and joint regimes of attention presuppose that shared cultural expectations among members of a social group are learned through immersive participation in social practices and become encoded neuronally as high-level priors [7,9]. The newly emergent acceptance of modernist art could be seen as a case study of a cultural practice that configured specific regimes of attention, resulting in the enculturation—the enskillment of an ever-growing community of art viewers. It would be possible to draw on historical research to fill in the crucial details of the story of modern art acceptance and canonization: for example, a growing number of (modern art) exhibitions were held, they generated a positive response, and this in turn attracted more people; museum education began to have an influence on the public; the reflection of modern art in the mass media led to ever-larger audiences. Nevertheless, the above-mentioned models are very abstract, and despite such theoretical proposals there is at present no viable conceptual model of joint PE minimization.^3^ At any rate, the mechanisms of PEM cannot fully explain the why and how of enculturation processes [105].

## 7. Conclusion

This paper presented a case study of how the PC framework can provide a mechanistic account of the cognition and behaviour that underlie certain phenomena studied by the history and sociology of art. It argues that at the root of two well-documented types of incomprehension of art—(i) the incomprehension of contemporary conceptual and minimal art and (ii) the incomprehension of early modern avant-garde art—are two different modes of a failure of the optimal PEM that is involved in the perception of art. I suggest that the ability to recognize pictorial content in modernist painting, which is the prerequisite for the wider acceptance of this art, required an updating of a number of expectations in order to optimize the fit between priors and sensations—from low-level perceptual priors to the development of higher-level, culturally determined expectations. Admittedly, given the available space, the focus here was exclusively on visual predictions, while art experience is also substantially shaped by interoceptive and somato-visceral predictions. To advance this account it will be necessary to bridge the gap between single person PEM in perception and the understanding of art and its propagation on a society-wide level. Predictive processing has been postulated as an overarching paradigm of brain function, linking perception, cognition and behaviour. Perhaps the arts, as one of the most complex manifestations of the human mind, will increasingly test its ambition to provide a unifying account of mental functioning.

## Data Availability

This article has no additional data.

## Endnotes

1 That is not to say that works labelled as conceptual art cannot provide cognitively challenging and ultimately satisfying experiences. For instance, using the example of Duchamp's work, Gregory Minissale has demonstrated how the interplay of visible and invisible aspects of work prompt extended cognitive engagement [13].

2 The case studies presented here are necessarily simplified as the temporal dynamics of error reduction are left out. In reality, both *Holes in Cardboard* and Marc's *Mandrill* would have been viewed not in isolation, but within the trajectory of a museum visit, during which other works would also have been encountered. Thus PEM in both cases would take place against the background of the viewer's implicit estimation of the overall rate of reduction of prediction errors over the course of an entire visit (and possibly the visitor's encounters with other works of art).

3 Or, as Karl Friston remarked, ‘…a lot of heavy lifting would have to be done to go beyond single-person prediction error minimisation’ (discussion during the conference *Art and Affect in Predictive* Mind, 1st July 2021.).
